# Characteristics of the School Food Environment Affect the Consumption of Sugar-Sweetened Beverages Among Adolescents

**DOI:** 10.3389/fnut.2021.742744

**Published:** 2021-10-08

**Authors:** Luana Lara Rocha, Milene Cristine Pessoa, Lúcia Helena Almeida Gratão, Ariene Silva do Carmo, Nayhanne Gomes Cordeiro, Cristiane de Freitas Cunha, Tatiana Resende Prado Rangel de Oliveira, Larissa Loures Mendes

**Affiliations:** ^1^Preventive and Social Medicine Department, Universidade Federal de Minas Gerais, Belo Horizonte, Brazil; ^2^Nutrition Department, Universidade Federal de Minas Gerais, Belo Horizonte, Brazil; ^3^Pediatrics Department, Universidade Federal de Minas Gerais, Belo Horizonte, Brazil; ^4^Ministry of Health, Brasília, Brazil; ^5^Nutrition Department, Pontifícia Universidade Católica de Minas Gerais, Belo Horizonte, Brazil

**Keywords:** food environment, school, sugar-sweetened beverage, adolescent, public health

## Abstract

Sugar-sweetened beverages are widely available and accessible in school environment, and their presence and characteristics of this environment can influence their consumption. This study examines the association of drinking fountains per 100 students, soft drink sales, soft drink advertising, and the presence of street vendors and sugar-sweetened beverages consumption among adolescents in Brazil. This cross-sectional study was carried out using data from the Study of Cardiovascular Risk in Adolescents that was conducted between March 2013 and December 2014. The sample comprised 71,475 adolescents aged 12–17 years from 1,247 public and private schools in Brazilian cities. Sugar-sweetened beverages consumption was the dependent variable. The main effect was the school food environment, which was evaluated based on drinking fountains per 100 students, soft drink sales, soft drink advertising, and the presence of street vendors. Public and private schools that sold soft drinks were associated with higher average sugar-sweetened beverages consumption among adolescents. Our study highlights the importance of creating healthy school food environments by banning sugar-sweetened beverages in schools accordingly.

## Introduction

Sugar-sweetened beverages (SSB) are ultra-processed beverages, as they are industrial formulations composed of antioxidants, stabilizers, and preservatives (1). In Brazil, the Food and Nutritional Surveillance System identified that in 2019, 65% of adolescents (*n* = 235,590) consumed SSB the day before the interview (2). Excessive consumption of SSB by adolescents has been associated with them being overweight and obese (3–7), the incidence of chronic non-communicable diseases (8–10), and the incidence of mental disorders (11–14).

SSB are widely available and accessible in Brazil, including in the school food environment, which may promote the consumption of these beverages (15–18). The school food environment encompasses the space, infrastructure, and conditions within and outside the schools, where food is available, purchased, and consumed accordingly. This environment also involves the facility of available information on food and nutrition, promotion, and pricing of such foods (19).

Evaluating the characteristics of the school environment and its influence on the consumption of SSB is important for identifying the axes of action for public policies. Several researchers (20–26) recommend school environment regulations and food and nutritional education actions to reduce the availability, purchase, advertising, and consumption of these beverages. The World Health Organization (WHO) published a document in 2016 with a series of recommendations for reducing soft drink consumption among children and adolescents. The main recommendations of this document concerning the school food environment were to increase the availability of potable water in schools, conduct food, and nutritional educational programs to make children and adolescents aware of healthy beverage options, reduce the availability of sweetened beverages in the school environment, and ban marketing of sweetened beverages in schools (20).

The school environment has been extensively researched as a place where adolescents are present for a large part of their day and is considered an important determinant for food choices, contributing to the consumption of unhealthy foods (18, 27–32). International studies have found that SSB availability in the school food environment is associated with the purchase and consumption of these beverages by children and adolescents (27, 33–36). However, in Brazil, there is only one study that evaluates the association between soda consumption and its availability in school cafeterias and canteens (21). This is the first study to identify, in a broader way, the association between the characteristics of the school food environment and SSB consumption among Brazilian adolescents.

Thus, the objective of this study is to examine the association between the characteristics of the school food environment and SSB consumption among Brazilian adolescents. This study can help strengthen the scientific evidence that justifies the implementation of the regulation of the sale of unhealthy food in the school environment. However, regulations on the sale of unhealthy foods and beverages already exist in some Brazilian cities and states. These regulations are not extended to the entire country and schools, and hence, a more effective regulatory measure is needed to cover all Brazilian schools.

## Methods

### Study Population

A cross-sectional study was carried out using data from the Study of Cardiovascular Risk in Adolescents (Portuguese acronym, “ERICA”), that was conducted between March 2013 and December 2014. The ERICA study was a cross-sectional, national, and school-based study that aimed to estimate the prevalence of cardiovascular risk factors and metabolic syndrome among adolescents aged 12–17 years who attended public and private schools in Brazilian cities with a population of >100,000 (37). Bloch et al. (38) and Vasconcellos et al. (37) have provided detailed information regarding the sampling process, research protocol, and data collection.

In this study, we examined adolescents from 1,247 schools in 124 Brazilian municipalities. The research population was stratified into 32 geographical strata: 26 state capitals, 1 federal district, and 5 strata representing other municipalities in each macro region of the country. Schools were selected based on the number of students, and the probability was inversely proportional to the distance between the non-capital municipalities and the state capital. Three classes were selected per school with different combinations of school schedule times (morning and afternoon) and grades (seventh, eighth, and ninth grades of elementary school; first, second, and third grades of high school). All students in the selected classes were invited to participate in the study. Adolescents not in the age group of 12–17 years, with some degree of physical disability that hindered them from undergoing the anthropometric assessment, and pregnant adolescents were not eligible to participate.

The ERICA sample consisted of 102,327 eligible adolescents, of which 73,160 responded to a 24-h food recall, and 74,589 completed the self-administered questionnaire (available for download at: http://www.erica.ufrj.br/index.php/metodos/) using a personal digital assistant, model LG GM750Q (consisting of 105 questions divided into 11 blocks, covering sociodemographic, health, and lifestyle aspects). Therefore, adolescents who had complete data for both the adolescent questionnaire and 24-h food recall were examined accordingly (39).

Most adolescents who did not participate in ERICA, because they were not present on the day or refused to participate, were male and aged 15–17 years. The adolescents participating in ERICA did not differ statistically from those who did not participate in the study (39).

ERICA was approved by the Research Ethics Committees of the Institute of Studies in Collective Health of the Federal University of Rio de Janeiro in each state of Brazil and the Federal District. All adolescents who agreed to participate provided written informed consent. When the local ethics committee required informed parental consent, such consent was obtained from the students to participate in the study.

### Food Consumption Assessment

Food consumption was assessed using a 24-h food recall through a face-to-face interview performed by trained interviewers, who used the Brazil Nutri software to record the food consumption data with a direct recording of information on netbooks. The interview technique used was the multiple-pass method (40), which consisted of an interview guided in five stages that intended to reduce the under-reporting of food consumption. The software used contained a list of 1,626 food items from the database regarding the acquisition of food and beverages from the 2002 to 2003 household budget survey conducted by the Brazilian Institute of Geography and Statistics (IBGE) (41), which was developed by the Ministry of Health.

### Dependent Variable

In this study, SSB consumption (mL/day) was the dependent variable. This variable was obtained from the sum of the quantities of the following types of drinks consumed by adolescents: regular soda, energy drinks, industrialized fruit juices (powdered and carton fruit juices), and milk-based beverages with added sugar and chemical additives. Adolescents reporting daily consumption of >3,000 mL of SSB were excluded from the sample to eliminate outliers that may have been caused by measurement errors; thus, finally 71,475 adolescents were included in the study. The adolescents who were excluded did not significantly differ from the other adolescents who were included in the study, considering other analyzed variables.

### Main Effect

The main effect was the school food environment, which was evaluated based on drinking fountains per 100 students, soft drink sales, soft drink advertising, and the presence of street vendors. To collect data from the school environment, a systematic social observation was conducted by trained researchers using a previously designed questionnaire. This questionnaire consisted of 28 questions about the physical structure, availability of physical education teachers, school meals, and food sales.

The drinking fountains per 100 students were obtained by dividing the number of drinking fountains by the total number of students enrolled in the school, multiplied by 100 students. The sale of soft drinks was obtained by accounting for all types of soft drink sales within the school, whether through canteens or self-service machines, respectively. Soft drink advertising and the presence of street vendors in the 100 meters around the school (only those who sold soft drinks were considered) were obtained through direct observation.

### Adjustment Variables

The variables that were used to describe the sample and as adjustment variables in multilevel linear regression models included sex, self-reported skin color, age, socioeconomic score, school region, offering of school meals, and proximity to the capital (if the school was located in the capital or inner-state city).

The socioeconomic score was defined using the 2013 Brazilian Economic Classification Criterion (CCEB) of the Brazilian Association of Research Companies (ABEP) (42), in which the possession of assets (colored television, radio, bathroom, automobile, refrigerator, freezer, washing machine, and DVD player), presence of a domestic worker, and education of the head of the family was considered (42). However, in 30.8% of the questionnaires, no information on maternal education was obtained. Thus, we opted for the use of “wealth proxy,” as adopted by Moura (43), and renamed this variable as the socioeconomic score, that considered only the possession of assets and the presence of a domestic worker. Thus, rather than analyzing the economic class, the socioeconomic score was categorized into three equal intervals (low: 0–12, medium: 13–25, and high: 26–38).

### Statistical Analyses

Descriptive analysis included the calculation of relative frequencies, medians, and percentiles, stratified by the type of school (public or private). Multilevel linear regression models were used to investigate the association of drinking fountains per 100 students, soft drink sales, soft drink advertising, and the presence of street vendors with consumption of SSB, stratified by the type of school (public or private). Unstandardized regression coefficients (β) and 95% confidence intervals (95% CI) were used as a measure of effect.

For Supplementary Material, we used Student's *t*-test, analysis of variance, and Bonferroni test to compare the means of SSB consumption between the categories of each variable. To compare the averages between public and private schools, we used Student's *t*-test. Parametric statistical tests were performed on account of the sample size.

The analyses were performed using Stata software (version 14.0; StataCorp LP, College Station, USA). Notably, in all the performed analyses, the sample complexity was considered using the Stata svy command, with a significance level of 5%.

## Results

A total of 71,549 adolescents and 1,247 schools were evaluated in this study. The prevalence of SSB consumption was 68.3% among adolescents from public schools and 74.6% among adolescents from private schools, respectively (considering adolescents who consumed any amount of SSB on the day evaluated using a 24-h food recall).

Table 1 shows the characteristics of the adolescents according to the type of school. Adolescents from public schools were predominantly female, brown-skinned, aged 14–15 years, and were classified as having a medium socioeconomic level. Adolescents from private schools were predominantly male, white-skinned, aged 12–13 years, and were classified as having a high socioeconomic level.

**Table 1:** Characterization of Brazilian adolescents evaluated by the ERICA study, Brazil (2013–2014; *n* = 71,549).

**Variables**	**School type**	
	**Public**	**Private**
**Sex (%)**		
Female	50.2	47.91
Male	49.8	52.09
**Race/skin color (%)**		
White	37.1	54
Black	9.3	3.8
Brown	50.8	39.4
Yellow	2.1	2.2
Indigenous	0.7	0.6
**Age (years) (%)**		
12–13	32.6	47.2
14–15	37.1	24.8
16–17	30.3	28
**Socioeconomic Score (%)**		
High	17.4	52.7
Medium	79.8	46.8
Low	2.8	0.5

Table 2 shows the characteristics of the school environment according to the type of school. Public schools predominantly do not sell soft drinks, do not have advertising for soft drinks, do not have a street vendor around the school, and have a median of 0.3 drinking fountains per 100 students. Private schools predominantly sell soft drinks, do not have advertising for soft drinks, do not have a street vendor around the school, and have a median of 0.9 drinking fountains per 100 students.

**Table 2:** Characterization of Brazilian schools evaluated by the ERICA study, Brazil (2013–2014; *n* = 1,247).

**Variables**	**School type**	
	**Public**	**Private**
**Soft Drinks Sale (%)**		
No	64.4	23.9
Yes	35.6	76.1
**Soft Drink Advertising (%)**		
No	96.8	86
Yes	3.2	14
**Street Vendor (%)**		
No	75.7	82
Yes	24.3	18
Drinking fountains per 100 students *(median, P25–P75)*	0.3 (0.2–0.5)	0.9 (0.5–1.6)

The mean of SSB consumption by adolescent consumption between the categories of each variable is provided in the Supplementary Material.

The school food environment variables associated with SSB consumption in the multiple generalized estimation equation models are shown in Table 3. Public and private schools that sell soft drinks are associated with higher average SSB consumption among adolescents.

**Table 3:** Association between sugar-sweetened beverages consumption and the school environment among Brazilian adolescents included in the ERICA study, Brazil (2013–2014; *n* = 71,549).

**Variables**	**Sugar-sweetened beverages consumption (mL)**			
	**Public school**		**Private school**	
	**β (95% CI)^**1**^**	**β (95% CI)^**2**^^,^ ^a^**	**β (95% CI)^**1**^**	**β (95% CI)^**2**^^,^ ^b^**
Drinking fountains per 100 students	1.5 (−20.2; 23)	−6.2 (−26.2; 14)	2 (−7.3; 11)	1.5 (−7.4; 10.3)
Soft drinks sale	16 (−2; 33.4)	17.5 (2; 33)^*^	26.2 (−3.2; 56)	55 (24; 86)^**^
Soft drink advertising	−9 (−55.3; 37)	5.2 (−35.4; 46)	−16 (−51.4; 19.4)	−10 (−44.3; 21)
Street vendor of soft drink	−5 (−24.3; 14.2)	8.8 (−8; 26)	−15.5 (−48; 17)	−11 (−42; 21)
*Variance*	13,147 (11,756.2; 14,702)	8,919 (7,889; 10,083)	6,072 (4,661; 7,909.2)	5,205 (3,916.2; 6,918)
*Intraclass correlation coefficient*	0.08 (0.07; 0.09)	0.06 (0.05; 0.07)	0.4 (0.3; 0.6)	0.04 (0.03; 0.05)
*Akaike criteria*	765,332.5	700,726	207,450.5	190,126.2

*CI, Confidence Interval; mL, milliliters.*

1 *Unadjusted multilevel linear regression model.*

2 *Adjusted multilevel linear regression model.*

a *Adjusted for gender, race/ethnicity, age, socioeconomic score, school region, offer school meals, and capital.*

b *Adjusted for gender, race/ethnicity, age, school region, and capital.*

* *p < 0.05.*

** *p = 0.001.*

*Values in bold are statistically significant (p < 0.05)*.

## Discussion

It was possible to evaluate the association between the characteristics of the school food environment and SSB consumption in this cross-sectional study of Brazilian adolescents. It is important to emphasize that the consumption of these beverages with high energy density and low nutritional value causes damage to adolescent health (5, 6, 8, 9, 11–14), and a reduction in their consumption has been hence recommended (20).

Concerning the school's characteristics, public and private schools that sold soft drinks were associated with higher average SSB consumption in adolescents. Other studies outside Brazil have also evaluated the association between school characteristics and SSB consumption and found that the presence of vending machines or school cafeterias was related to a higher average consumption of these beverages for adolescents (27, 33, 34). Richardson et al. found that the availability to purchase unhealthy foods and beverages in schools was positively associated with the purchase of SSB by adolescents in the United States (36).

In Brazil, our study is the first to examine the association between the characteristics of the school food environment and adolescents' SSB consumption in a broader way. The results of our study followed the same trend as observed in international studies (27, 33–36). The availability and sale of SSB in the school environment was associated with a higher average consumption of these beverages by adolescents because they were cheaper, readily available, and hyper-palatable (20).

Evaluation of the school environment's impact on the consumption of SSB stratified by the type of school is important because of the differences in these environments. In a study using data from ERICA, Carmo et al. (16) found that the food environment of private schools has obesogenic characteristics owing to the sale and advertising of ultra-processed foods.

Public schools also have a protective factor against the consumption of ultra-processed food. On account of the food provided by the National School Feeding Program (Programa Nacional de Alimentação Escolar [PNAE]) (17, 21, 44). PNAE offers free and healthy food and provides nutrition education to all students from federal, state, municipal, and district public schools; it also prohibits the purchase of beverages with low nutritional value (soft drinks, artificial refreshments, beverages, or concentrates based on guarana or currant syrup, ready-to-drink teas, and other similar beverages) (45). The presence of these school feeding programs has contributed to the promotion of healthy eating among children and adolescents in several countries (46, 47). These programs are a tool in promoting a healthy school food environment.

However, the presence of soft drink sales in and around public schools is associated with the consumption of SSB. Although the PNAE provides the necessary food for adolescents during school time, the presence of a vendor that sells ultra-processed food and beverages has a negative effect on the healthy food environment provided by the national program. Previous studies have shown that the presence of food and beverage sales in schools was associated with an increase in the chances of SSB and ultra-processed food consumption (16, 48). In addition, the presence of this type of sale was associated with a reduction in the chances of consuming school meals provided by the PNAE (49).

It is important to emphasize that actions alone are not sufficient to deal with the decrease in SSB consumption. The consumption of these beverages is associated with many complex factors, which involve not only the food environment, but also the culture, food preferences, social circle, and other factors (50–53). Therefore, only the implementation of the PNAE will not be enough to reduce the consumption of sweetened beverages, and other measures are necessary, such as the implementation of regulatory measures, food and nutritional education actions, and support from parents and family (20, 23, 26, 54).

For private schools, it was also found that the sale of soft drinks was associated with higher average SSB consumption among adolescents. This result suggests that, regardless of the school type, having food and beverage sales promotes unhealthy eating habits in the school environment, by increasing the consumption of ultra-processed food and beverages. Furthermore, private schools in Brazil are at a disadvantage because they already have a more obesogenic food environment and are not covered by the PNAE. This is so because regulatory measures that normalize the sale of food in the school environment are directed only to public schools (16, 22, 55).

The importance of adopting regulatory measures that prohibit the sale of SSB in and around public and private schools should be highlighted accordingly. This measure has been recommended by several researchers (20–23) and has proven to be effective (56, 57). It is important to emphasize that these regulatory measures do not prohibit the sale of food in general. They aim to prohibit only the sale of ultra-processed foods, such as soft drinks, industrialized juices, isotonic, package snacks, and cookies, among other foods that are considered industrial formulations. Presence of natural and minimally processed foods should be encouraged in the school environment.

In Brazil, some cities and states have local laws restricting unhealthy food and beverage sales inside schools, but the effectiveness of these regulatory measures is uncertain, and the vast majority do not include private schools (22, 58, 59). Therefore, a national law is needed to regulate the sale of unhealthy food and beverages that cover public and private schools as well as their surroundings, reinforcing the importance of a healthy food environment for healthy food consumption. Concerning SSB only, Law Project 1,755 of 2007 has the objective of banning the sale of soft drinks in canteens of basic education schools. This Law Project is ready to be voted on by the House of Representatives. However, there is no indication that this law will enter the voting agenda. In Brazil, it is difficult to approve measures that directly affect the ultra-processed food and beverage industries. These companies influence the state, which is the cause of political inertia in regulatory measures aimed at ultra-processed food and beverages.

This study has some limitations. A 24-h food recall does not have a great characterization of the actual habitual consumption among adolescents and can lead to bias in the dietary assessment, including misreporting, underestimating, or overestimating the consumption of SSB for atypical days. However, the use of the multiple-pass method and the representative sample (71,533 adolescents) reduces the chance of errors and increases the reliability of 24-h food recall (40, 60). Although statistically significant, they were low with respect to the magnitude of the associations. It is important to note that this result is due to the more distal influence of the food environment on food consumption. Moreover, we have no information on where SSB were consumed; we only know that they are being consumed, making it impossible to quantify SSB consumption in the school.

The strengths of this study are (1) the representative sample that allows generalization in the Brazilian population of adolescents aged 12–17 years and residing in cities with >100,000 inhabitants, and (2) this is the first study to investigate the association between the characteristics of the school food environment and SSB consumption among Brazilian adolescents. In addition, this study highlights the importance of creating healthy school food environments by banning SSB in schools.

## Data Availability Statement

The datasets presented in this article are not readily available because the data underlying this study are from ERICA (http://www.erica.ufrj.br/) and were provided by author Dr. Cristiane De Freitas Cunha who is the coordinator of the study at the state of Minas Gerais (http://www.erica.ufrj.br/index.php/equipe/). Future researchers can request to access the same data using the information provided in the Materials and Methods section of the article and applying for access on the ERICA website or by emailing projetoerica@gmail.com. Requests to access the datasets should be directed to http://www.erica.ufrj.br/index.php/equipe/ or ERICA Project, projetoerica@gmail.com.

## Ethics Statement

The studies involving human participants were reviewed and approved by Institute of Studies in Collective Health of the Federal University of Rio de Janeiro and the Research Ethics Committees of each state of Brazil and the Federal District. Written informed consent to participate in this study was provided by the participants' legal guardian/next of kin.

## Author Contributions

LR and LM idealized the article. LR, LG, and NC wrote the manuscript. LR and AC performed the analyzes and designed the methodology. CC, TO, MP, and LM were responsible for the database and designed the methodology. All authors reviewed and commented on subsequent drafts of the manuscript.

## Conflict of Interest

The authors declare that the research was conducted in the absence of any commercial or financial relationships that could be construed as a potential conflict of interest.

## Publisher's Note

All claims expressed in this article are solely those of the authors and do not necessarily represent those of their affiliated organizations, or those of the publisher, the editors and the reviewers. Any product that may be evaluated in this article, or claim that may be made by its manufacturer, is not guaranteed or endorsed by the publisher.
