# Supplementary material for: Characteristics of the School Food Environment Affect the Consumption of Sugar-Sweetened Beverages Among Adolescents
Source: Front Nutr. 2021 Oct 8;8:742744. doi: 10.3389/fnut.2021.742744 (PMC8531082; doi:10.3389/fnut.2021.742744)
Supplement: Supplementary file 1 [file Table_1.DOCX]

**Table S1.** Sugar-sweetened beverages consumption among Brazilian adolescents evaluated using the ERICA study, Brazil (2013–2014; n=71,549)

| **Variables** | **Sugar-sweetened beverages consumption (mL)¹** | | | | |
| --- | --- | --- | --- | --- | --- |
|  | **Public school** | | **Private school** | | **p-value**** |
|  | **Mean, SD** | **p-value*** | **Mean, Sd** | **p-value*** |  |
| *Gender* |  | |  | |  |
| Female | 320 (371) | **<0.001** | 307 (348) | **<0.001** | **0.01** |
| Male | 364 (431) |  | 367 (405) |  | 0.6 |
| *Race/Skin color* |  | |  | |  |
| White | 368 (410)^a,b^ | **<0.001** | 332 (374) | 0.06 | **<0.001** |
| Black | 354 (414)^c,d^ |  | 372 (402) |  | 0.3 |
| Brown | 322 (389)^a,c^ |  | 336 (378) |  | **0.01** |
| Yellow | 341 (413) |  | 301 (334) |  | 0.09 |
| Indigenous | 289 (343)^b,d^ |  | 347 (344) |  | 0.2 |
| *Age (years)* |  | |  | |  |
| 12–13 | 329 (383)^a^ | **<0.001** | 339 (366)^a^ | **<0.001** | 0.08 |
| 14–15 | 347 (403)^a^ |  | 346 (383)^b^ |  | 0.8 |
| 16–17 | 339 (406) |  | 316 (378)^a,b^ |  | **<0.001** |
| *Socioeconomic score* |  | |  | |  |
| High | 405 (443) ^a,b^ | **<0.001** | 332 (382) | 0.8 | **<0.001** |
| Medium | 331 (391) ^a,c^ |  | 335 (371) |  | 0.5 |
| Low | 256 (348) ^b,c^ |  | 313 (360) |  | 0.2 |

SD: standard deviation

Note: equal letters represent statistically significant difference.

¹Milliliters

*t-test or ANOVA (comparison between categories of the variable)

**t-teste (comparison between types of school)

**Table S2.** Sugar-sweetened beverages consumption among Brazilian schools’ characteristics evaluated using the ERICA study, Brazil (2013–2014; n=1,247)

| **Variables** | **Sugar-sweetened beverages consumption (mL)¹** | | | | | |
| --- | --- | --- | --- | --- | --- | --- |
|  | **Public school** | | **Private school** | | |  |
|  | **Mean, SD** | **p-value*** | **Mean, SD** | **p-value*** | **p-value**** | |
| *School region* |  | |  | | |  |
| North | 293 (389)^a,b,c,d^ | **<0.001** | 296 (368) ^a,b,c,d^ | **<0.001** | 0.8 | |
| Northeast | 278 (346)^a,e,f,g^ |  | 326 (364)^a,e,f^ |  | **<0.001** | |
| Southeast | 381 (405)^b,e,h^ |  | 342 (367)^b^ |  | **<0.001** | |
| South | 450 (446)^c,f,h,i^ |  | 361 (409)^c,e^ |  | **<0.001** | |
| Midwest | 371 (431)^d,g,i^ |  | 355 (398)^d,f^ |  | 0.1 | |
| Capital |  | |  | | |  |
| No | 353 (408) | **<0.001** | 362 (392) | **<0.001** | 0.3 | |
| Yes | 334 (396) |  | 329 (373) |  | 0.2 | |
| *Offers school meals* |  | |  | | |  |
| No | 282 (377) | **<0.001** | - | - | - | |
| Yes | 340 (399) |  | - |  | - | |
| Soft drinks sale |  | |  | | |  |
| No | 334 (394) | **<0.001** | 317 (377) | **<0.01** | **0.02** | |
| Yes | 349 (408) |  | 339 (376) |  | **0.03** | |
| *Soft drink advertising* |  | |  | | |  |
| No | 339 (398) | 1.0 | 335 (377) | 0.3 | 0.3 | |
| Yes | 339 (407) |  | 326 (373) |  | 0.3 | |
| *Street vendor* |  | |  | | |  |
| No | 339 (399) | 0.9 | 336 (376) | 0.06 | 0.6 | |
| Yes | 339 (397) |  | 321 (375) |  | **0.03** | |

SD: standard deviation

Note: equal letters represent statistically significant difference.

¹Milliliters

*t-test or ANOVA (comparison between categories of the variable)

**t-teste (comparison between types of school)
